# The Wear and Tear of Modern Life

**Published:** 1886-08

**Authors:** 


### SELECTIONS.

### The Wear and Tear of Modern Life.

A discussion on the above subject, recently initiated by Dr. Roose, in the *Fortnightly Review*, has been largely taken up by the press, and treated in a manner befitting its importance. There has long prevailed a conviction, the outcome in part of intuition, in part of evidence, which, in its nature is somewhat indefinite, that we are living at too fast a rate, and that the pressure shows signs of increase rather than of diminution.

There seems no reason to dispute the prevalent impression that nervous diseases are commoner than formerly, and that we suffer vastly more than our forefathers from the sense of pressure, worry, and unrest. Mr. Goschen, in his recent admirable address on hearing, reading, and thinking, remarked with too certain truth that not only are we always in a hurry, but that we would be ashamed to admit that it was ever otherwise. This high tension, though no doubt peculiarly characteristic of metropolitan life, prevails more or less in all our large centres of population, and we cannot regard its possible further increase without apprehension, both for ourselves and those who will come after us. To a certain extent, it is the inevitable outcome of modern civilization. Steam has not merely quickened physical movement, but its accelerating effect has operated in every department of life and manners. The telegraph and telephone mark the victory of the nineteenth century over space and time, but we pay for the boon in an increased expenditure of vital energy. Education has enlarged its scope and its claims at so rapid a rate, that already the timid are beginning to demand a halt.

Now, we are far from thinking that civilization will arrest its onward rareer or abate its claims, no matter how loud or how earnest be the notes of alarm. We cannot put the drag on human progress, or modify at will the complicated conditions of modern society. Ease of existence and simplicity of manners are not to be obtained by merely sighing for them. But, while we can do little to stay the onward relentless rush of modern progress, it is worth our while to inquire what are its peculiar dangers, and whither it is surely tending. We cannot leap from the express train, but it is something to know whither we are going, and whether the road is clear.

Medical science offers us this preliminary consolation. Hard work, physical or mental, is not only not hurtful, but is actually healthful. The hard-worked muscle, wheather of arm or heart, responds to the stimulus, and receives an accession of vigor and and development. The hard-worked brain, if placed under favorable conditions for its activity, becomes more vigorous and fruitful. Indolent inactivity of body or mind is undoubtedly far more injurious to both physical and intellectual vigor than severe labor, provided only that the labor be pursued under healthful conditions of food, air, rest, and sleep. This, in brief, is the message of science, that not labor, but its too often abnormal environment, is responsible for the deterioration of the physical and intellectual powers. The factory operative who succumbs at forty, dies not of hard work, but of foul air and defective diet. Overpressure in the schools means, for the most part, not that the brain is getting too much food, but that the stomach is getting too little. The student induces brain-weariness, not merely by excessive study, but by late hours, insufficient sleep, lack of fresh air, and perhaps, an undue partiality for the tobacco-leaf or the tea-pot. It cannot be too strongly proclaimed, that the end of modern civilization is, not that we should arrest the pressure of work, which it is unnecessary to do, and, if necessary, would be impossible, but that we should learn to work under healthful and rational conditions.

One of the most genuine difficulties of modern life lies in the hindrances which exist to the proper adjustment of work. The hard worker is the man who maintains a high average of work, not he who crowds into one day the toil of two, and then spends two or three days of idleness in attempting to recuperate his powers. No error is more frequent or more disastrous than this. Much vexation, disappointment, and disease would be averted, if we would ascertain the limits of our powers, and realize that to exceed them is not an economy of time, but precisely the reverse. This is a hard lesson to learn, and it is sometimes learnt too late. *Si jeunesse savait et si vieillesse pouvait*—if youth only knew how rightly to use its powers, and if age only retained the powers which it learns, perhaps too late, how to use! This attempt to crowd into one day the work of two has the grave disadvantage of entailing a great expenditure of vital force as the result of worry. We wear ourselves out, not by hard work, but by anxious thought regarding the adjustment of work, and by nervous irritation at the failure to accomplish an impossible task. A complex social system makes multiplicity of claims upon us; but, if we would live wisely, we must learn so to adjust those claims, that we shall discharge each in the order of its importance, and not waste time and energy in constant hesitation as to which has the right of priority.

Science gives us a further consolation in its dictum, that rest is not to be found in inertness, but in change of occupation. The over-worked man often looks forward to his annual holiday, and promises himself the great luxury of simply doing nothing. He tries the experiment, and, in nine cases out of ten, it is a disastrous failure. He dreams of lying on his back, and watching the clouds or the sea, and enjoying a delicious indolence. He gets his wish, and he is more weary and miserable than ever before. He has forgotten that he is no longer a child, and that he has lost forever the capacity of being idle. But a distinct change of occupation would have given him the distraction which he needs. Thus, we see a great statesman spending his leisure of the Parliamentary recess in maintaining a philosophic controversy, or a great naturalist turning aside to discourse upon the choice of books. Amid the high tension of the nineteenth century, it is some consolation to know that the weary brain-worker need not seek absolute idleness; but, by turning aside into fresh pastures, may find new material for the healthful exercise of that mental activity which has become second nature.

The conclusion at which we arrive is, then, that the need of our age is not rest and stagnation, but healthful conditions for work, freedom from worry, suitable variety, and a wise distribution of our time.

In the April number of the *Fortnightly Review*, Dr. Roose resumes the thread of his former discourse, and indicates some of the remedies for the "wear and tear" which formed the subject of this first article. He enlarges with perspicacity and good sense upon food, rest, sleep, recreation, and kindred topics. On some points—for example, the value of horseback exercise to the brain-worker, the importance of rest not only after but before eating, and the necessity of seeing that the annual holiday affords mental distraction as well as physical repose—we can strongly endorse the opinion of the writer. Dr. Roose's articles are apt and timely, and they are sober and practical; they express opinions generally entertained by thoughtful medical men, and are well worth the attention of the wider circle of busy brain-workers, who require from us, not a prohibition of work, but help and guidance towards its better and easier accomplishment.—*The British Medical Journal.*

